# Atmospheric kraft pulping in glycerol

**DOI:** 10.1038/s41598-026-61930-8

**Published:** 2026-07-11

**Authors:** Carl Moser, Mikael Ghaysari, Gunnar Henriksson

**Affiliations:** Department of Fibre and Polymer Technology, Wallenberg Wood Science Center, KTH Royal Institute of Technology, 100 44 Stockholm, Sweden

**Keywords:** Kraft pulping, Organosolv pulping, Pressure, Glycerol, Biorefinery, Chemistry, Energy science and technology, Engineering, Environmental sciences, Materials science

## Abstract

Kraft pulping is the predominant technique for producing chemical pulps from wood. It offers low operational costs, versatility, high-quality pulps, and an efficient closed chemical recovery system. However, the process requires significant investment due to the necessity for pressurized environments and the associated costly equipment. This study explores a novel approach by replacing water with glycerol, allowing kraft pulping to occur at atmospheric pressure. However, the higher viscosity of glycerol compared to water partially limits impregnation. This research investigates the impact of temperature, chemical charge, and chip size on this process for both hardwood and softwood sapwood. The results indicate that lignin dissolution is expedited by reducing chip size for both softwood and hardwood. However, decreasing the chemical charge negatively impacts lignin dissolution. Conversely, increasing the temperature accelerates lignin release.

## Introduction

 The predominant method employed in the pulp and paper industry for producing chemical pulp from wood pulp is the kraft pulping process^1^. This process involves treating wood chips in a solution of sodium hydroxide (NaOH) and sodium hydrogen sulfide (NaSH) at elevated temperatures^2^. The primary objective is to separate the fibers from the wood matrix by degrading and dissolving lignin^3^. The process is relatively flexible with respect to raw material, although the required cooking severity differs between hardwood and softwood. The resulting fibers are strong and suitable for papermaking^4^. However, the kraft process is associated with significant yield losses due to the degradation and removal of lignin and carbohydrates, especially at high temperatures and chemical charge (alkali)^5^. Running costs for kraft pulping are often moderate due to its well-designed chemical and energy recovery system^6^. Kraft pulping requires high temperatures and must be conducted at elevated pressures when water is used as the solvent. This necessitates using expensive equipment, such as digesters, resulting in substantial investment costs for pulp mills. To offset these costs, there has been a trend toward large-scale production facilities to justify significant capital investment. Consequently, new kraft mills are generally extensive and often involve continuous processes^7^. From a bio-economical perspective, this presents challenges, as many industrial lignocellulose byproducts (e.g., straw, corn stover, rice husks and shells) do not generate large enough volumes to sufficiently sustain traditional kraft mills^8^.

Although kraft pulping has its advantages, ongoing research is being conducted into alternative processes for chemical pulping, such as processing lignocellulosic biomass in organic solvents (organosolv). Certain organosolv methods have proven effective in separating the various components of wood, enabling further processing into a range of different products^9,10^. However, most of these solvents have boiling points lower than water, and the associated processes require substantial investments.

In contrast, Linden et al.^11^ explored the use of glycerol as a processing medium under atmospheric pressure, facilitated by glycerol’s high boiling point. This approach simplifies experimental setups by enabling continuous sampling without the need for an autoclave, allowing reactions to be conducted in a standard fume hood with real-time analysis of chemical consumption. Additionally, the elimination of high-pressure vessels provides industrial benefits, including potential cost savings and a reduced risk of pressure-related hazards. Beyond the experimental convenience, the atmospheric system offers unique opportunities for real-time process monitoring and detailed studies of impregnation and delignification behavior. Its open configuration allows for the integration of in-line analytical tools, such as UV-Vis spectroscopy for lignin concentration, pH and conductivity sensors for alkali tracking, and potentially Raman or NIR spectroscopy for carbohydrate degradation. These capabilities make the system an attractive model for understanding reaction kinetics and liquor penetration under controlled conditions, which are difficult to achieve in conventional pressurized digesters. These findings highlight glycerol’s promise not only as a practical lab-scale medium but also as a potentially sustainable alternative for future biorefinery applications.

In this study, atmospheric pressure refers to the cooking stage itself. Vacuum impregnation was examined as a pre-treatment to assess whether the limited impregnation associated with the high viscosity of glycerol could be improved. It is therefore not proposed as a required element of the process, but as a tool for evaluating impregnation limitations and possible process trade-offs.

While the results are promising, further work is needed to assess the scalability of the process. Investigating how processing parameters such as chip size, wood species, and chemical charge affect impregnation efficiency and product quality will be essential to optimize the system and expand its industrial relevance. While this study explores the potential technical advantages of atmospheric kraft pulping in glycerol, the economic implications discussed are speculative and intended to highlight possible directions for future research. A comprehensive techno-economic analysis, including capital and operational expenditures, solvent recovery, and energy demands, lies beyond the scope of this work.

This study aimed to develop a lab-scale pulping process using glycerol in a fume hood, highlighting the advantages of this method for both practical and industrial applications.

## Materials and methods

### Materials

**Fig. 1:**
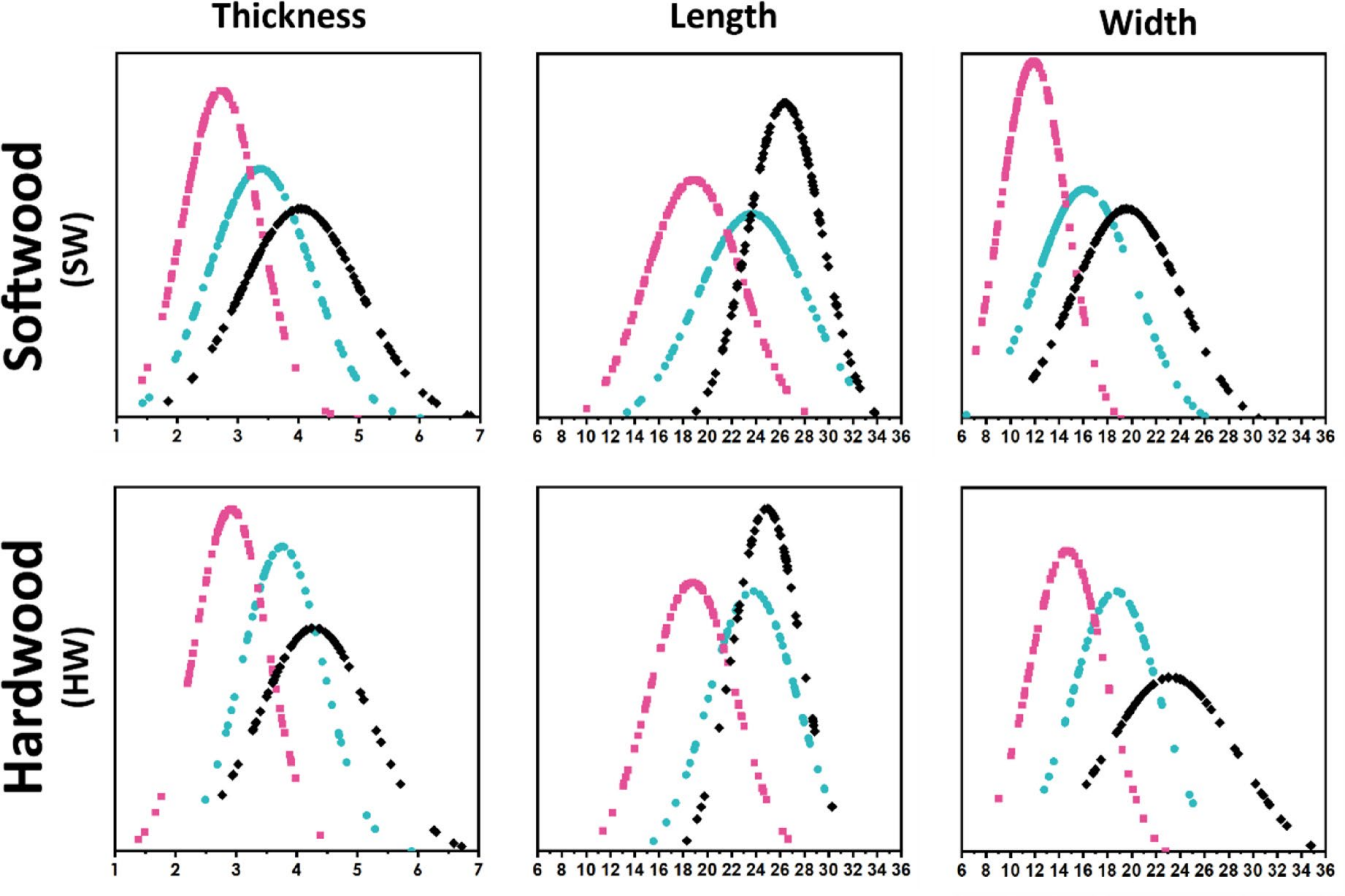
is a 2 × 3 matrix of plots showing size distribution for the different sizes of chips. Rows: Softwood (top), Hardwood (bottom). Columns: Thickness, Length, Width. X‑axes show size in millimeters; Y‑axes show normalized frequency. In each panel, three curves represent chip‑size fractions (Square marker) ■ for small “S”, (circular marker) ● for medium “M” and (diamond marker) ♦ for large “L”. In each panel, S peaks at smaller x‑values than M, and M peaks at smaller x‑values than L. Thus, within a species, moving from S to M to L shifts the distribution to the right for thickness, length, and width. Patterns are qualitatively similar for Softwood and Hardwood.

Chips from Norway spruce (*Picea abies*) and birch (*Betula pendula* or *Betula pubescens*) sapwood were obtained from single trees sourced from (63° 48’ 43.210” N, 20° 14’ 9.564” E, Sundsvall, Sweden)^12^. The material was supplied by the Swedish University of Agricultural Sciences (SLU, Umeå), where species identification was verified by qualified personnel. The trees were harvested from privately owned land. No voucher specimens were deposited, as the material was obtained as wood chips from a controlled source and not collected for taxonomic purposes. The collection and use of plant materials complied with relevant institutional and national regulations and did not involve protected or endangered species. The wood was chipped at a pilot-scale chipper (Bruks Mekaniska AB, Sweden). Wood chips (“A”) were manually sorted into three different fractions, Small (S), Medium (M) and Large (L), and approximately 100 chips of each size were measured using a caliper with an accuracy of 0.01 mm (Table 1). The size distribution of softwood and hardwood chips differs across thickness, length, and width, with clear separation among small, medium, and large fractions (Fig. 1).

**Table 1:** Chip-size averages for hand-sorted chips of Softwood (SW) and Hardwood (HW).

	Thickness (mm)	Length (mm)	Width (mm)	Area (cm3)	Weight per chip (mg)
SWS	2.73 ± 0.12	18.89 ± 0.75	11.91 ± 0.51	0.61	141.1
SWM	3.37 ± 0.16	23.69 ± 0.86	16.14 ± 0.77	1.29	275.9
SWL	4.04 ± 0.18	26.38 ± 0.57	19.58 ± 0.84	2.09	513.5
SWA	3.38 ± 0.11	23.00 ± 0.54	15.88 ± 0.54	1.23	309.0
HWS	2.92 ± 0.11	18.77 ± 0.66	14.70 ± 0.60	0.80	305.2
HWM	3.76 ± 0.13	23.75 ± 0.69	18.69 ± 0.68	1.67	581.5
HWL	4.29 ± 0.17	24.89 ± 0.53	23.25 ± 1.00	2.48	906.4
HWA	3.60 ± 0.14	22.21 ± 0.64	18.60 ± 0.81	1.49	578.2

Unsorted chips “A” is the average of all measured chips for each species. “S” refers to the smallest fraction of chips, “M” medium fraction, and “L” large fraction.

Glycerol of GPR Rectapur grade (VWR International), NaOH of Emsure grade from Sigma-Aldrich and sodium hydrosulfide hydrate from Sigma-Aldrich were used.

### Atmospheric processing

A stock solution was prepared by dissolving NaOH (1 M) and NaSH (0.21 M) in glycerol in a volumetric flask with a magnetic stirrer at a temperature of 60 °C. Processing was carried out in 100 mL round-bottom flasks. Each bottle was filled with 100 mL (126 g) stock solution and a magnet and heated to the impregnation temperature (See Table 2 for all processing parameters). Once at target temperature, 10 g chips (dry content = 94.6%) for a liquor to wood ratio of 12:1 were added to the flask and kept in place beneath the surface using a metallic wire mesh. For experiments that included vacuum impregnation (Table 2), a vacuum was introduced and controlled for 15 min to facilitate deeper impregnation of the pulping liquor into the biomass. The removal of air from the cell lumen improves liquid penetration during subsequent processing steps^13^. Once the allotted impregnation time (120 min for birch, HW, and 180 min for spruce, SW) had elapsed, the temperature was increased to the processing temperature. Aliquots of 3 mL were collected throughout the reaction (180 min for birch, HW, and 240 min for spruce, SW).

**Table 2:** Processing parameters for birch (HW) and spruce (SW).

Sample	Imp. temp. (°C)	Processing temp. (°C)	NaOH (M)	NaSH (M)	Vacuum (Yes/No)	Chip size (A/S/M/L)
SW(S/M/L)	140	160	1	0.21	No	S/M/L
HW(S/M/L)	130	150	1	0.21	Yes	S/M/L
HWA_(V)_	130	150	1	0.21	Yes/No	A
HWA_L_	120	140	1	0.21	No	A
HWA_H_	140	160	1	0.21	No	A
HWA_0.75_	130	150	0.75	0.16	No	A
HWA_0.5_	130	150	0.5	0.11	No	A

Unsorted chips “A” is the average of all measured chips for each species. “S” refers to the smallest fraction of chips, “M” medium fraction, and “L” large fraction. High and low processing temperature is denoted by “_H_” and “_L_” respectively. The introduction of a vacuum impregnation is denoted by “_V_”. Chemical charge is denoted as the percentage of standard, “_0.75_” and “_0.5_” respectively.

The experiments were designed as comparative process series rather than a full factorial design. Selected parameter combinations were used to examine the effects of chip size, vacuum impregnation, chemical charge, and impregnation/processing temperature under practically relevant conditions. Therefore, some variables were changed together, and the results are interpreted within each experimental series. In particular, the chip-size comparisons for hardwood and softwood were performed under different impregnation conditions, so the chip-size effects are interpreted within each species-specific experimental series.

After processing, glycerol black liquor was collected, and the chips were washed with deionized water using a flow-through washing funnel, allowing the dissolved lignin to leach out of the chips. Pulp was produced by subjecting the cleaned chips to a disintegrator, after which the shives were separated by a modified BDDJ (Britt Dynamic Drainage Jar) setup.

Kappa analysis was conducted following ISO 302:2004, which involves permanganate oxidation followed by iodometric titration. This method quantifies the residual lignin in pulp, providing an indicator of the degree of delignification achieved during pulping.

Intrinsic viscosity was determined according to ISO 5351:2010 by dissolving oven-dried pulp in cupriethylenediamine (CED) solution and measuring flow time in a calibrated capillary viscometer at 25 °C. The limiting viscosity number was calculated and expressed in mL/g.

For comparison, two reference samples were prepared using conventional kraft pulping in aqueous media under pressurized conditions. Softwood reference (SWR) was cooked at 170 °C with an effective alkali (EA) charge of 18% and sulfidity of 40%. Hardwood reference (HWR) was cooked at 157 °C with EA 18% and sulfidity 36%. These conditions represent standard industrial kraft pulping parameters for spruce and birch, respectively. The resulting pulps were analyzed using the same methods as described above for yield, kappa number, brightness, and viscosity (see Table 3).

### Black liquor analysis

The soluble lignin content was measured by determining the absorbance at 280 nm with UV-Vis spectroscopy using a Shimadzu UV-2550 spectrophotometer. The absorptivity increases as a linear function of the amount of lignin^14^. Samples were diluted to obtain values of A_280_ < 1. The concentration of lignin was calculated by utilizing Beer-Lambert’s law (1)^15^:

1 $$\:{\mathrm{C}}_{\mathrm{U}\mathrm{V}}=\frac{{\mathrm{A}}_{280}}{\epsilon\:\:\bullet\:\:\mathrm{l}}$$

Where *A*_280_ is the absorbance at 280 nm, *ε* is the extinction coefficient of the lignin in *g*^−1^*cm*^− 1^ and l is the length of the cuvette in cm. The extinction coefficient for spruce was assumed to be *ε*_*SW*_ *=* 25 *g*^−1^*cm*^− 1^ and for birch *ε*_*HW*_ *=* 13 *g*^−1^*cm*^− 1^^16^. Since the sample is diluted, the concentration *C*_*UV*_ is multiplied by a dilution factor *d*_*f*_. With this factor considered, Eq. 1 can be rewritten as Eq. 2 where *C*_*s*_ is the concentration of lignin in g/L. The concentration can then be plotted as a function of time.

2 $$\:{\mathrm{d}}_{\mathrm{f}}\bullet\:{\mathrm{C}}_{\mathrm{U}\mathrm{V}}=\frac{{\mathrm{A}}_{280}}{\epsilon\:\:\bullet\:\:\mathrm{l}}={\mathrm{C}}_{\mathrm{S}}$$

### Light optical microscopy

Optical microscopy was performed using a Leica DMIL LED microscope equipped with a 4×/0.10 Hi PLAN objective. Images were captured with a Leica D2900 camera and processed using LAS-X software. The thickness of fibers was measured across 50 points, and the length at 10 points.

## Results and discussion

**Table 3:** Results for varying wood species and processing parameters.

Sample	Yield (%)	Shives (%)	Kappa	Brightness (%)	Viscosity (mL/g)
SWS	48.7 ± 1.1	0.17 ± 0.11	14.2 ± 2.2	32.9 ± 0.6	1057
SWM	49.3 ± 1.5	1.24 ± 0.19	17.7 ± 0.1	33.5 ± 0.1	1122
SWL	48.6 ± 1.3	3.48 ± 0.87	19.9 ± 1.7	34.5 ± 0.2	1158
HWS	50.4 ± 0.9	0.38 ± 0.29	8.8 ± 0.1	34.2 ± 0.7	1252
HWM	50.0 ± 0.7	2.45 ± 0.46	10.5 ± 0.1	32.7 ± 0.9	1281
HWL	49.1 ± 0.8	6.37 ± 0.10	11.7 ± 0.1	33.2 ± 1.2	1278
HWA	44.1 ± 0.1	11.61 ± 0.94	13.7 ± 0.25	34.0 ± 0.5	-
HWA_V_	46.7	6.94	11.8	33.8 ± 0.3	-
HWA_L_	32.9	29.12	22.1	29.2 ± 0.1	-
HWA_H_	49.9	2.16	9.3	31.5 ± 0.1	-
HWA_0.75_	40.1	20.38	19.9	29.0 ± 0.2	-
HWA_0.5_	28.9	41.78	-	24.4 ± 0.1	-
SWR	43.5	0.97	23.9	33	1111
HWR	50.7	1.01	14.7	37.5	1269

The average value with standard deviation is shown for duplicate samples. Unsorted chips “A” is the average of all measured chips for each species. “S” refers to the smallest fraction of chips, “M” medium fraction, and “L” large fraction. High and low processing temperature is denoted by “_H_” and “_L_” respectively. The introduction of a vacuum impregnation is denoted by “_V_”. Chemical charge is denoted as the percentage of standard, “_0.75_” and “_0.5_” respectively. See Table 2 for a full breakdown of parameters.

The reference samples prepared by conventional kraft pulping showed properties broadly comparable to those obtained in glycerol-based atmospheric pulping (Table 3). In hardwood, the reference gave higher brightness than the atmospheric samples, despite having a similar or slightly higher kappa number, suggesting more effective removal of chromophoric structures under pressurized aqueous conditions.

The atmospheric pulps also reached low kappa numbers without a significant reduction in viscosity. This may indicate relatively limited carbohydrate degradation under the conditions studied, although viscosity is influenced mainly by cellulose degradation and may also be affected by hemicellulose dissolution and degradation.

### Effect of chip size

**Fig. 2:**
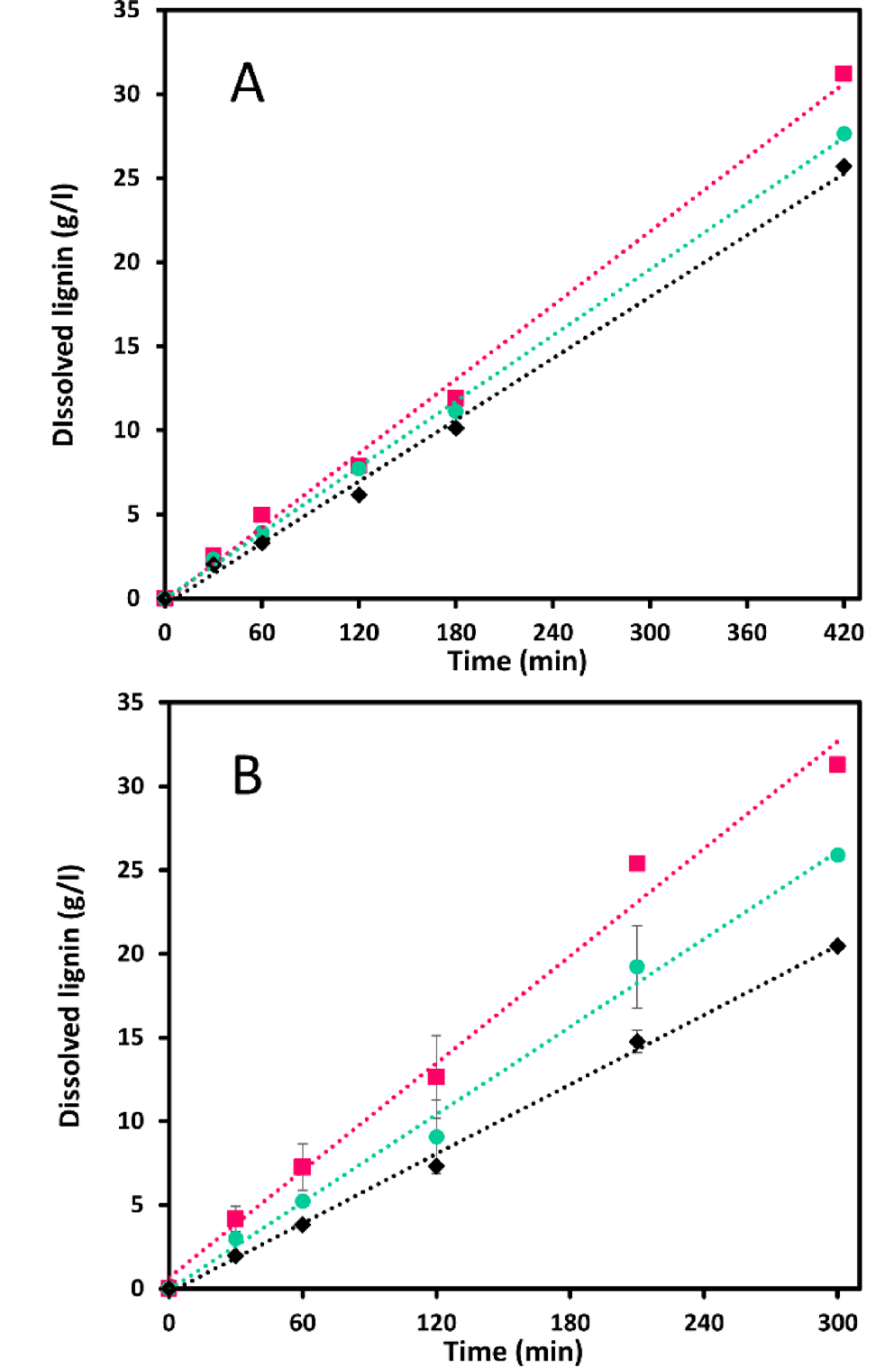
two separate scatter plots showing dissolved lignin (g/L) as a function of time for differently sized chips. Panel A (spruce, 0–420 min) and Panel B (birch, 0–300 min). Y‑axis runs 0–35 g/L in both. In each plot, three series represent chip sizes: squares ■ “S”, circles ● “M”, diamonds ♦ “L”. In both species, all three series rise nearly linearly from near zero to between 20–32 g/L by the final time point. At every time, S has the highest dissolved lignin, M is intermediate, and L is the lowest. Dotted linear fit lines closely match the data (R² = 0.99). Panel B includes vertical error bars representing duplicate measurements.

Both softwood and hardwood samples follow the expected trend: smaller chips release lignin more rapidly into solution. This is likely due to their larger surface area and thinner dimensions, which facilitate better impregnation. The effect is more pronounced in hardwood, possibly because these samples were subjected to vacuum impregnation, unlike the softwood. It should be noted that the chip-size series for hardwood and softwood were carried out under different impregnation conditions, since vacuum impregnation was applied only to the hardwood samples. The observed chip-size trends should therefore be interpreted within each species-specific series, and direct comparison of the magnitude of the chip-size effect between birch and spruce is limited.

For spruce (Fig. 2A), no clear differences in lignin release were observed among the S, M, and L chip fractions during the initial impregnation stage (0–180 min), even though smaller chips would be expected to delignify more rapidly; this suggests that impregnation was still limited during the early stage under these conditions. The linear increase in lignin concentration over time suggests a diffusion-controlled process. However, this is not conclusive, as delignification in viscous solvents, such as glycerol, involves multiple interacting factors, including chip geometry, evolving porosity, solvent penetration, and reaction kinetics. Although shorter diffusion paths in smaller chips may enhance lignin removal, the system is dynamic, and diffusivity and concentration gradients change throughout the pulping process. A non-linear trend, such as a plateau or exponential decay, would typically suggest saturation effects or changes in reaction kinetics over time. The absence of such behavior may reflect limitations in solvent penetration or a steady-state diffusion regime.

Table 3 shows that chip size does not significantly affect pulp yield, which is somewhat unexpected given the clear differences in shive content. Shives correlate well with chip size, and lower shives content could indicate overprocessing, which would typically result in reduced yield. The increase in shives content with chip size suggests incomplete delignification or poor impregnation in larger chips. However, the lack of corresponding yield reduction may indicate that shives are retained as part of the pulp mass, which could potentially affect downstream processing or product quality.

Viscosity measurements (Table 3) show a chip-size effect for softwood: larger chips exhibit higher viscosity (1158 mL/g) compared to smaller chips (1057 mL/g), suggesting less carbohydrate degradation in the former. Lower viscosity in small chips likely reflects overprocessing, resulting from better impregnation and shorter diffusion paths. In contrast, hardwood samples maintain consistently high viscosity (1252–1281 mL/g) regardless of chip size, indicating minimal cellulose damage under the applied conditions.

Kappa number follows the same trend as shives and dissolved lignin. Smaller chips yield pulp with lower kappa values, indicating more extensive removal of lignin. However, the differences in kappa number are less pronounced than those seen in shives and lignin concentration, with only an 11–25% increase observed when moving from small to large chips.

Brightness measurements do not clearly differentiate between samples. While there may be a slight trend in the spruce samples that align with shive content, kappa number, and lignin release, the overall brightness variation is minimal, at around 2%, and insufficient to draw strong conclusions.

**Fig. 3:**
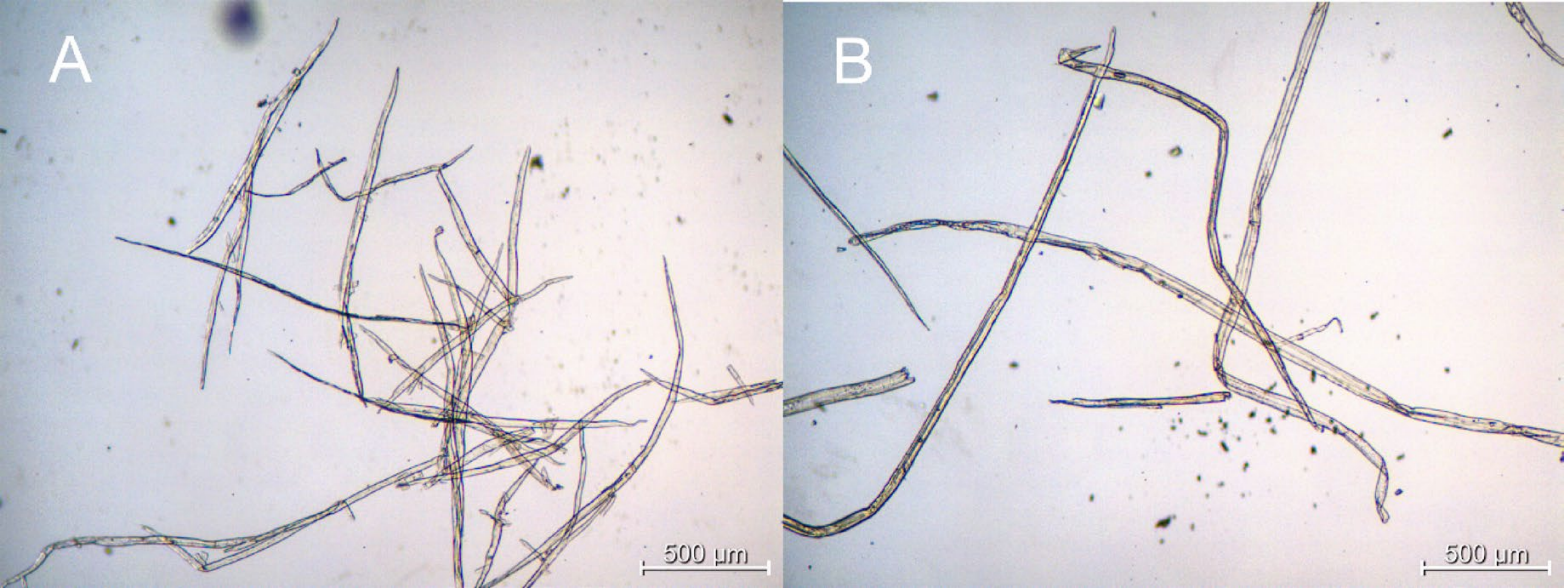
Two bright‑field micrographs shown side‑by‑side. A (HWM): several long, thin, semi‑transparent fibers extend diagonally across the field, overlapping near the center. B (SWM): a loose cluster of fibers occupies the center with additional isolated fibers radiating outward, fibers appear both longer and thicker than for the birch sample in. Scalebar shows 500 μm. Fibers had been dried prior to measurement, making them stiffer than their undried counterpart.

Light microscopy (Fig. 3) showed that the examined fibers remained largely intact after atmospheric pulping. However, these observations are qualitative and based on limited sampling. For birch fibers (Fig. 3A), the average thickness was 24 ± 2 μm, with an approximate length of 1.06 mm. Spruce fibers (Fig. 3B) showed an average thickness of 36 ± 2 μm and an approximate length of 3 mm. These measurements fall within the typical range reported in the literature and are positioned toward the upper end, indicating relatively high values compared to previously published data, with birch fibers averaging around 1 mm in length and 22 μm in width^17,18^, and spruce fibers averaging between 2.5 and 3 mm in length and 26–35 μm in width^19–21^.

The microscopy supports only the qualitative conclusion that no obvious fiber collapse or severe fragmentation was observed in the examined samples. More advanced techniques, such as fiber image analysis, staining, scanning electron microscopy, or mechanical testing, would be needed for a more rigorous assessment.

### Effects of vacuum impregnation

**Fig. 4:**
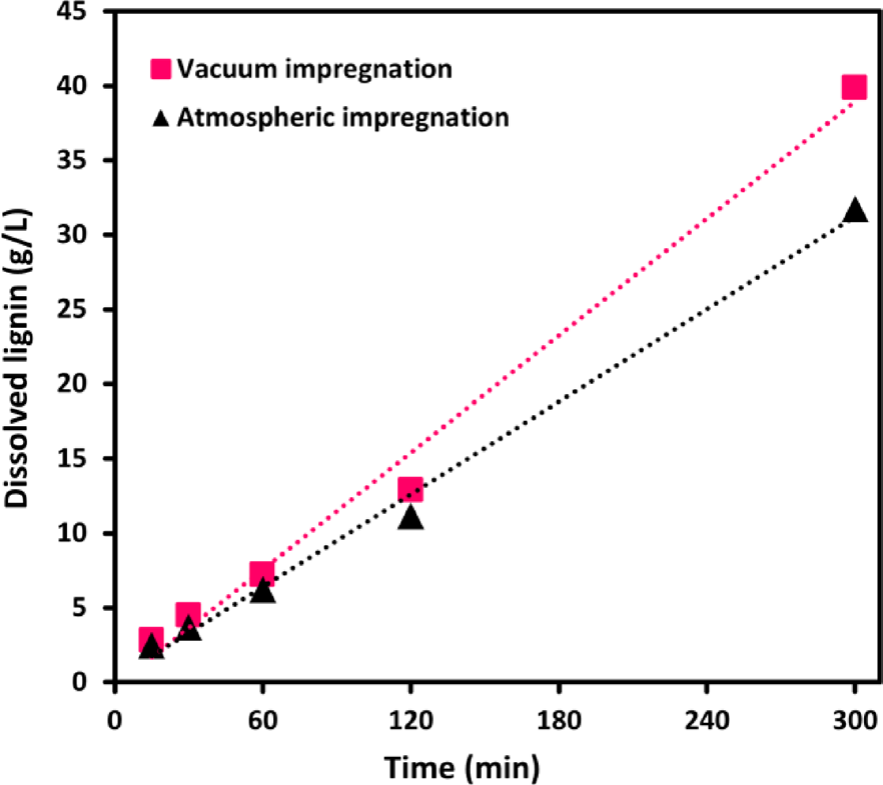
Influence of vacuum impregnation on the delignification of birch (HW). Chips were added to the stock solution for 18 h prior to processing. The time was measured from when the chip/stock solution mixture reached the impregnation temperature. Dotted lines are linear fits with r^2^ = 0.99.

Vacuum impregnation was introduced to improve penetration of the cooking liquor into the wood chips by removing entrapped air from the porous structure prior to pulping. As shown in Table 3, vacuum impregnation slightly increased yield, from 44.1% to 46.7%, while reducing shives content from 11.61% to 6.94% and lowering the kappa number from 13.7 to 11.8; brightness remained essentially unchanged. In Fig. 4, the difference in lignin release between vacuum and non-vacuum impregnation was small at earlier stages of the process and became more apparent only at longer processing times. The present data suggest that vacuum impregnation improved liquor penetration and pulping uniformity, but they do not clearly show at what stage the lignin dissolution rate increased.

Although vacuum impregnation improved birch pulping under the conditions studied, it should be regarded here as an optional pre-treatment rather than an inherent part of the atmospheric cooking concept.

### Effect of chemical charge

**Fig. 5:**
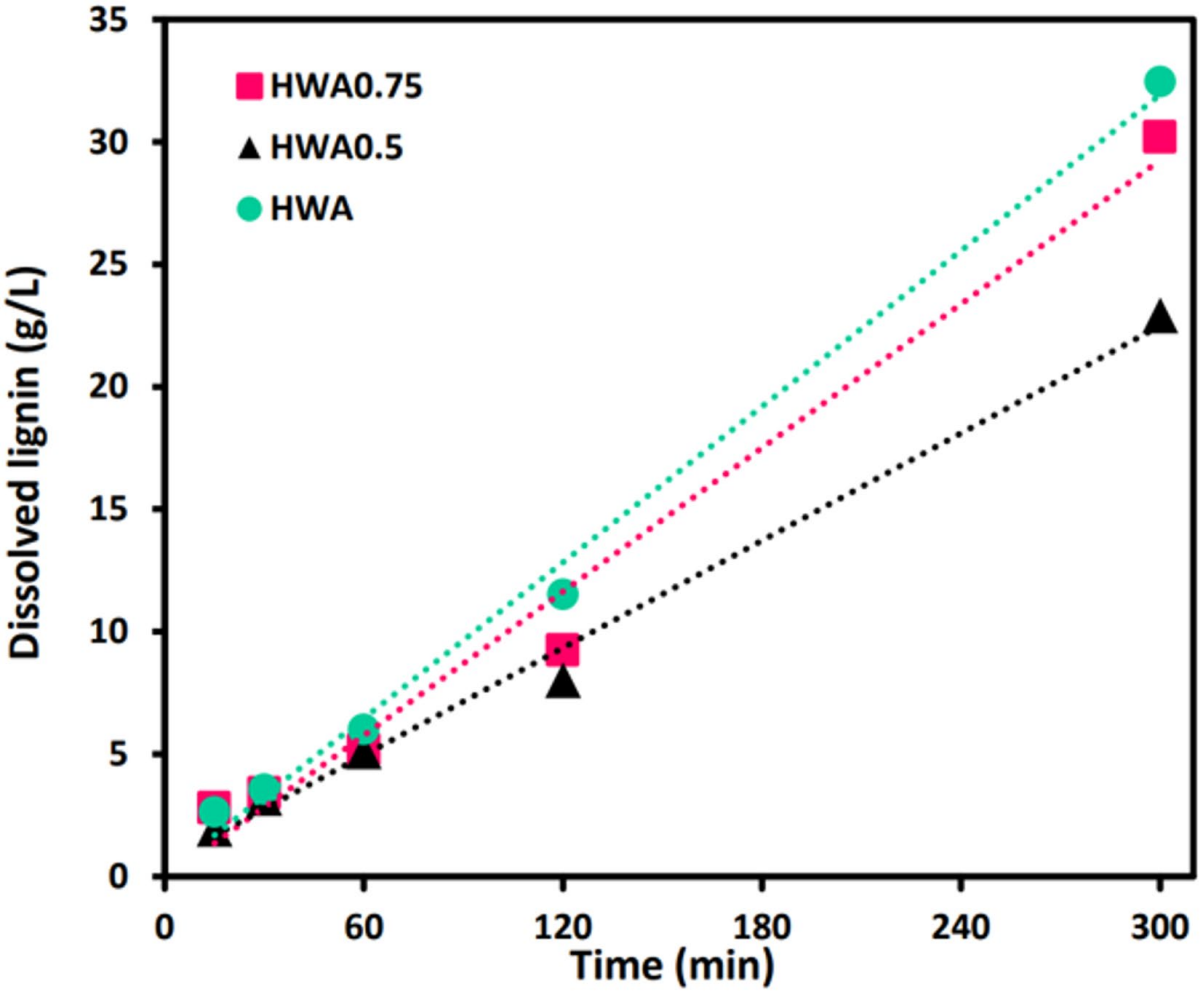
Influence of chemical charge on the delignification of birch (HW). Scatter plot of dissolved lignin versus time (0–300 min; 0–35 g/L). Three series: circles ● (HWA), squares ■ (HWA_0.75_), triangles ♦ (HWA_0.5_). All series rise linearly from near zero. Representative points: at 60 min, HWA = 6 g/L, HWA_0.75_ = 5 g/L, HWA_0.5_ = 5 g/L; at 120 min, HWA = 11 g/L, HWA_0.75_ = 9 g/L, HWA_0.5_ = 8 g/L; at 300 min, HWA = 32 g/L, HWA_0.75_ = 30 g/L, HWA_0.5_ = 23 g/L. Linear trendlines closely match the data (R² = 0.99).

In this series of experiments, only the chemical charge was altered while maintaining an impregnation temperature of 130 °C for 120 min, followed by processing at 150 °C for 180 min (Fig. 5).

For unsorted chips (HWA) yield decreased from 44.1% to 40.1% when decreasing the chemical charge to 75% (HWA_0.75_) of the native charge. This decrease was further exaggerated when decreasing the chemical charge to 50% reducing the yield to 28.9%.

A similar, but even more pronounced effect, is seen for the shives content, where the shives content increased from 11.61% for HWA to 20.38% for HWA_0.75_ and to 41.78% for HWA_0.5_. This signifies that the experiments with reduced chemical charge have not reached the defibration point, the most extreme example of this is HWA_0.5_ where the amount of shives is higher than the yield fibers.

Kappa follows a similar trend, going from 13.7 (HWA) to 19.9 (HWA_0.75_), indicating increased levels of lignin remaining in the pulp when reducing the chemical charge. Kappa could not be measured for HWA_0.5_ due to a lack of material.

Since the difference in processing was so pronounced even differences in brightness of the final pulp could be observed. Decreasing from 34% for HWA, to 29% for HWA_0.75_ to 24.4% for HWA_0.5_, further verifying the high levels of lignin remaining in the pulp associated with the low yields.

### Effect of impregnation and processing temperature

**Fig. 6:**
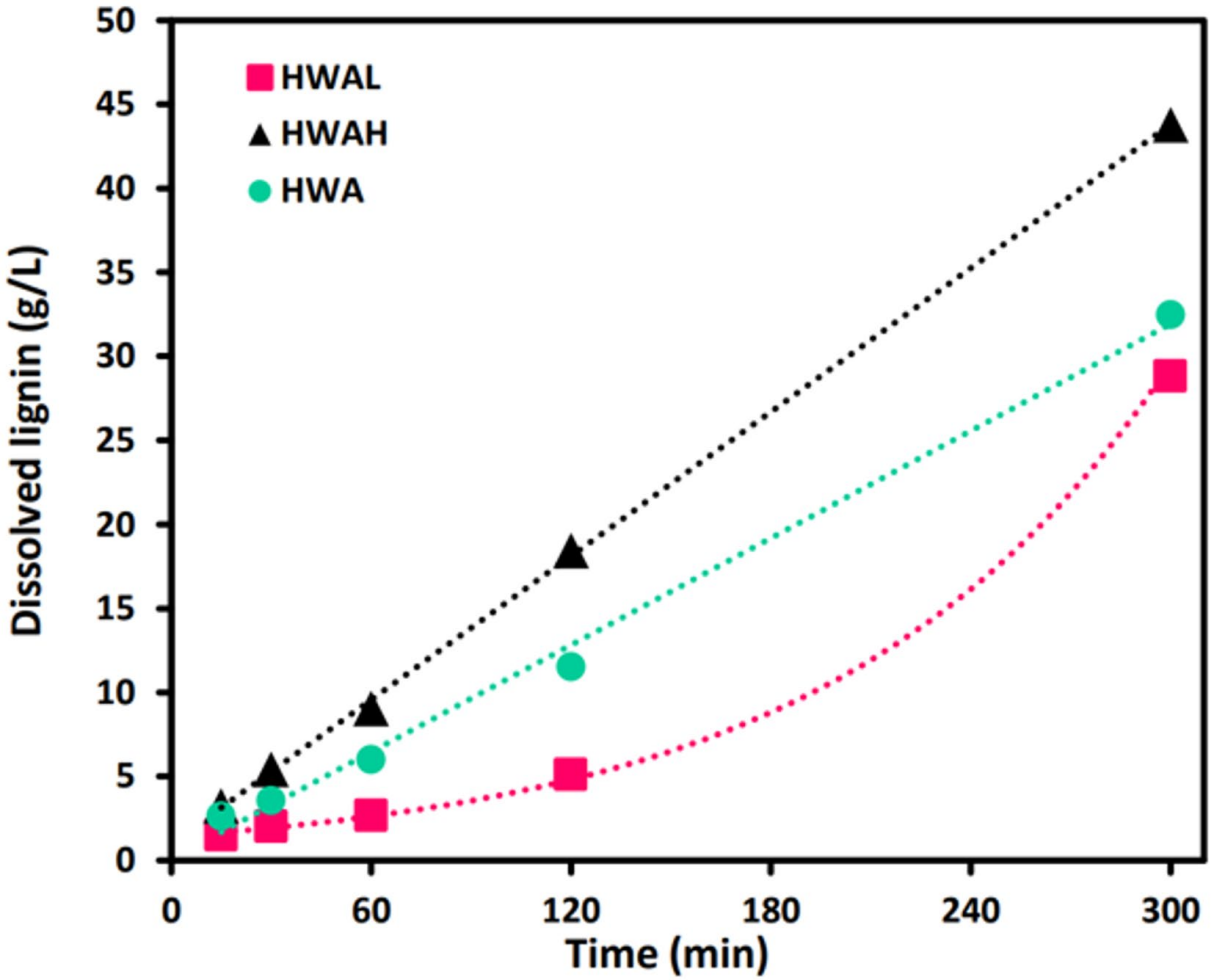
Influence of impregnation and processing temperature on the delignification of birch (HW). Dotted lines are linear fits with r^2^ = 0.99 for HWA and HWA_H_ and an exponential fit with r^2^ = 0.99 for HWA_L_.

Because the impregnation and processing temperatures were varied together in this series, the results are discussed as the effect of the applied temperature conditions rather than temperature alone. Higher temperature conditions increased the rate of lignin release (Fig. 6). The lowest temperature (120 °C impregnation and 140 °C processing) exhibited an exponential increase in dissolved lignin in contrast to all other data. This could be explained by poor impregnation and poor mobility due to the high viscosity of glycerol at relatively low temperatures.

The highest yield (49.9%) was observed for the highest temperature HWA_H_ followed by the reference sample HWA (44.1%) and lowest temperature HWA_L_ exhibited a yield of 32.9%. This is further verified by the data for shives and kappa (Table 3). Shives content decreased from 29.12% for HWA_L_ to 11.6% for HWA and 2.16% for HWA_H_, indicating that the lowest-temperature condition had not yet reached the defibration point. Kappa number followed the same trend, decreasing from 22.1 for HWA_L_ to 13.7 for HWA and 9.3 for HWA_H_.

### Technical significance

The use of atmospheric pressure may reduce the need for pressure-rated equipment, potentially lowering capital costs in specific applications. However, this potential advantage must be weighed against the added complexity of solvent recovery systems, and any benefit from vacuum impregnation must likewise be balanced against the added equipment and operating complexity. Atmospheric impregnation therefore remains the simpler baseline option from a process-integration perspective.

WThe system can be optimized to accommodate annual crops and residual biomass by adjusting parameters such as particle size, impregnation conditions, and residence time, thereby offering a versatile platform for decentralized processing of diverse lignocellulosic feedstocks. This flexibility is particularly relevant for underutilized materials like straws and stalks, which are often locally abundant but challenging to process using conventional methods due to their morphology and lower density.

While glycerol is often described as a relatively inexpensive and abundant byproduct of the biodiesel and oleochemical industries, its cost must be considered in context. Global glycerol prices range between USD 600 to 800 per ton, depending on the region and purity. The conventional pulping medium, water, is nearly free in most industrial operations. Organosolv pulping generally requires a high solvent-to-biomass ratio (approximately 4:1 by weight), meaning that the production of one ton of pulp may require up to four tons of glycerol even for an optimized system. This results in a raw solvent cost that can exceed the market value of chemical pulp, typically ranging from USD 1000 to 1300 per ton. These figures underscore the critical importance of efficient solvent recovery to ensure economic feasibility. Furthermore, global glycerol production is estimated to be between 2.9 and 4.4 million tons per year^22^, while global chemical pulp production exceeds 180 million tons annually^23^. Even if all available glycerol were redirected to pulping, it would support less than 2% of the current global pulp production. This clearly illustrates that large-scale deployment of glycerol pulping is only realistic if highly effective and economically viable recovery systems are in place.

Glycerol recovery is a crucial factor in determining the economic viability of this process. Due to glycerol’s high boiling point (290 °C), distillation requires a significant amount of energy input, likely involving high-grade heat sources such as direct combustion or steam. A realistic recovery system would involve multiple unit operations:

- **Lignin precipitation**, acidification of spent liquor using CO₂ gas to precipitate lignin. This also produces sodium salts (Na₂CO₃ and NaHCO₃), which can be regenerated into NaOH by calcination.
- **Solid-liquid separation**, by centrifugation or filtration, to remove precipitated lignin. Possibly preheated to lower viscosity and increase separation speed.
- **Sodium salts separation**,** f**ollowing CO₂-induced precipitation of lignin, the solids fraction may contain sodium salts. These salts may co-precipitate or remain suspended due to the high viscosity of glycerol. To isolate them, sequential washing of the solid phase with minimal water or ethanol can selectively dissolve the salts while retaining lignin. Alternatively, solvent exchange with water may facilitate salt recovery via filtration and evaporation. Crystallization from the glycerol phase is theoretically possible but practically challenging due to viscosity and solubility constraints. These steps require further optimization to enable efficient chemical recovery.
- **Glycerol purification via Distillation**.
  - **Vacuum**, lowering pressure reduced the boiling point of glycerol, minimizing thermal degradation and energy demand.
  - **Thin film**, A thin film of liquid is spread over a heated surface, enhancing heat transfer and reducing residence time.
- **Sodium sulfide recovery.** In conventional kraft pulping, sodium sulfide (Na₂S) is regenerated via thermal reduction of sodium sulfate in a recovery boiler, forming a smelt that is dissolved to produce green liquor. This process is not applicable in our glycerol-based system due to the absence of combustion and smelt formation. While theoretical chemical reduction of Na₂SO₄ is possible, it requires high temperatures and specialized equipment. Alternatively, gas-phase recovery of hydrogen sulfide (H₂S) followed by alkaline absorption can regenerate NaSH; however, this approach is complex and safety-intensive. As such, sulfur chemical recovery is currently impractical in our system, and replenishment of NaSH from external sources may be necessary for continued operation^24^.

These steps are not trivial and would require careful engineering to ensure solvent purity, minimize losses, and maintain process safety. But this aspect remains largely unexplored and requires further investigation to assess its feasibility at larger scales and with repeated cycles.

Nevertheless, the system is straightforward to set up in a laboratory, and sampling is naturally simple. As such, it could serve as a useful tool for technical research in the field of chemical pulping.

## Conclusion

Kraft pulping of both hardwood and softwood was achieved in glycerol at atmospheric pressure, although the extent of delignification depended on chip size, impregnation conditions, chemical charge, and temperature. For both spruce and birch, smaller chips gave lower shives content and lower kappa number than larger chips, indicating improved impregnation and delignification. In birch, increasing the impregnation and processing temperature increased yield and reduced both shives content and kappa number, while decreasing the chemical charge had the opposite effect and strongly reduced pulping efficiency. Vacuum impregnation also improved birch pulping, although the effect was more evident at longer processing times than during the initial stage. Overall, the results show that glycerol enables kraft pulping at atmospheric pressure, but also that limited impregnation remains a central challenge in the system.

## Data Availability

The datasets used and/or analyzed during the current study are available from the corresponding author upon reasonable request.
